# What is visible across the visual field?

**DOI:** 10.1093/nc/niab006

**Published:** 2021-06-01

**Authors:** Andrew M Haun

**Affiliations:** Center for Sleep and Consciousness, Department of Psychiatry, University of Wisconsin-Madison, WI, USA

**Keywords:** computational modeling, contents of consciousness, perception, psychophysics, peripheral vision, colour perception

## Abstract

It is sometimes claimed that because the resolution and sensitivity of visual perception are better in the fovea than in the periphery, peripheral vision cannot support the same kinds of colour and sharpness percepts as foveal vision. The fact that a scene nevertheless seems colourful and sharp throughout the visual field then poses a puzzle. In this study, I use a detailed model of human spatial vision to estimate the visibility of certain properties of natural scenes, including aspects of colourfulness, sharpness, and blurriness, across the visual field. The model is constructed to reproduce basic aspects of human contrast and colour sensitivity over a range of retinal eccentricities. I apply the model to colourful, complex natural scene images, and estimate the degree to which colour and edge information are present in the model’s representation of the scenes. I find that, aside from the intrinsic drift in the spatial scale of the representation, there are not large qualitative differences between foveal and peripheral representations of ‘colourfulness’ and ‘sharpness’.

## Introduction

When looking at a scene one may get the feeling that a visual experience is colourful and sharp across the full extent of one’s visual field. Even a savvy observer who knows about the higher objective resolution of foveal vision, or about the regular, rapid, and involuntary movement of the foveae from one part of the scene to another, is likely to get this feeling. However, it is frequently argued that this feeling is illusory. Such arguments—which tend to be part of larger philosophical or theoretical accounts of vision (Dennett 2005; Schwitzgebel 2008; Cohen *et al.* 2016; Lau and Brown 2019)—are always claimed to be based on physiological or psychophysical facts about visual perception: cone density declines with retinal eccentricity (Curcio *et al.* 1990), acuity declines even more rapidly and more severely for colour than for achromatic targets (Anderson *et al.* 1991), contrast sensitivity is poorer peripherally than foveally for most spatial targets (Robson and Graham 1981; Pointer and Hess 1989), and declines more severely for colour targets (Anderson *et al.* 1991). Therefore, it is argued, however, it seems (or seems to seem) to us, peripheral vision just cannot support the kinds of colour or sharpness percepts supported by foveal vision, and the only explanation for our subjective feelings is that they are illusory.

In contradiction of this argument, the present study shows that human spatial vision permits nearly invariant representation of colour and sharpness across the visual field. To demonstrate this invariance, I use a detailed model of human spatial vision. The model is constructed to reproduce known psychophysical patterns of human contrast perception. Importantly, the main feature of the model that allows it to reproduce these patterns is that its local scale of encoding varies across the extent of its visual field; apart from this variation in local scale, the structure of the model is independent of visual field position. The model is able to closely simulate the same facts that have sometimes been deployed to make claims about degraded peripheral visual experience: its contrast sensitivity and acuity decline with eccentricity, and much more severely for colour stimuli. The analyses that I carry out on the outputs of this model are therefore based directly in the empirical observations that have driven some of the misconceptions at issue.

When discussing properties like colourfulness and sharpness, I take these to be properties intrinsic to an observer’s visual experience. Of course, physical stimuli—which are different kinds of things than perceptual representations—can evoke visual representations like colour or sharpness via sensitive mechanisms of perception. It should be clear that, if colour and sharpness are representations intrinsic to an observer, they do not need to depend on exactly what stimulus evoked them; sensitivity may vary from mechanism to mechanism, but still the same perceptual representation can be evoked by different stimuli. Evidence for the intrinsic nature of colour includes the dissociation of stimulus properties from perceptual colour experiences as in colour constancy (Gegenfurtner *et al.* 2015), lightness and brightness illusions (Adelson 2000), colour afterimages and hallucinations (McCollough 1965), colour phosphenes (Dobelle and Mladejovsky 1974; Grusser 1995), and colour in dreams (Kahn *et al.* 1962). Evidence for a corresponding view of sharpness includes similar dissociations, such as in blur adaptation aftereffects (Webster *et al.* 2002), hallucinatory percepts with clear detail (Richards 1971), sharp-textured phosphenes (Nebel 1957; Tyler 1978), and experience of clear percepts in dreams (Rechtschaffen and Buchignani 1992).

Although the data on which the model is based are not from what we would typically consider studies of consciousness, readers should consider the following in interpreting the results. When a subject in a routine psychophysics task, such as a contrast-detection task, reports seeing a (retrospectively) suprathreshold target by selecting the correct response alternative, we reasonably assume that the subject was *conscious* of that target (violations of this assumption, known as *blindsight*, are difficult to obtain in normal vision). In consciousness studies, this assumption is usually confirmed by collecting more detailed responses, for example having subjects also indicate their confidence in having just consciously experienced a target. It is well-known that confidence is strongly correlated with sensitivity (Fleming and Lau 2014), so it would seem natural to conceptually link model sensitivity to conscious visibility, and the results of the present study may be interpreted in light of this link. However, visual experience involves more than just spatial qualities: we also experience the perceptual organization of these qualities (Peterson and Kimchi 2013), and we may recognize them as grounding or constituting objects. Whether or not we experience spatial qualities in the absence of perceptual organization or recognition is a matter of debate (Block 2007; Schwitzgebel 2007; Simons 2007; Lau and Brown 2019), and cannot be addressed by the current study; in either case, the results of the present study are relevant to the *visibility*, if not the conscious experience, of spatial qualities.

## Methods

The spatial vision model was implemented in MATLAB (code available at https://osf.io/8xf9w/). Empirical data used to set model parameters, or for other purposes (e.g. the perceived blur analysis in Section Attention), was extracted from the original study papers using the online WebPlotDigitizer tool (Rohatgi 2017).

### Properties of spatial vision

Listed below are psychophysical properties directly relevant to the question of colour/sharpness perception across the visual field (some ‘exemplar’ references are provided for each, but all of these properties have been observed in numerous studies).

- Contrast sensitivity is relatively independent between the three opponent chromatic axes (the three ‘colour channels’: luminance-contrast, blue-yellow contrast, and red-green contrast). Put another way, interactions between same-channel patterns are much stronger than interactions between different-channel patterns (Krauskopf *et al.* 1982; Buchsbaum and Gottschalk 1983; Mullen and Sankeralli 1999).
- Spatial frequency acuity (the spatial frequency *f_a_* at which contrast sensitivity is = 1, i.e. minimal) is inversely proportional to eccentricity plus a constant (E_2_):*f_a_* = *f* * *E*_2_/(*E* + *E*_2_) (Strasburger *et al.* 2011).
- Foveal acuity is better for achromatic targets than for chromatic targets, and the proportionality constant *E*_2_ is higher (acuity declines less with eccentricity for achromatic than for chromatic targets) (Noorlander *et al.* 1983; Anderson *et al.* 1991, 2002).
- The high-spatial frequency decline in contrast sensitivity at any eccentricity is exponential (Yang *et al.* 1995): *S*(*f*) ∼ *n^−f^*. This holds over all colour channels (Fig. 10).
- Contrast sensitivity for a target of any spatial frequency declines exponentially with eccentricity, with a steeper exponent for higher spatial frequencies (Pointer and Hess 1989; Anderson *et al.* 1991): *S*(*E*) ∼ *n^−E^*. This also holds over all colour channels (Fig. 10).
- The visual system is low-pass and sensitivity across the visual field converges for very low spatial frequencies (Pointer and Hess 1989).
- Contrast sensitivity (as d’) for targets of increasing contrast follows an expansive/compressive power function (threshold-vs-contrast functions are dipper-shaped) (Nachmias and Sansbury 1974; Legge and Foley 1980).
- ‘Contrast constancy’: In the absence of other interactions, contrast responses converge at high contrasts, for mechanisms tuned to different spatial frequency, orientation, and/or eccentricity (Georgeson and Sullivan 1975; Cannon 1985; Swanson and Wilson 1985; Chen *et al.* 2000).
- Sensitivity for a low-contrast target of one orientation is strongly impaired by a high-contrast overlaid mask of very different orientation (‘cross-orientation masking’), while a high-contrast target is relatively unaffected by a lower-contrast mask (Foley 1994).
- The combined perceptual response to contrast over multiple frequency bands is a high-p-norm (less-than optimal combination: *M*∼ = 4) (Cannon and Fullenkamp 1991).

### Threshold over eccentricity and scale

Properties i-x are interrelated in various ways. Of particular importance, ii., iv. and v. (regarding relation of sensitivity with scale and eccentricity) are distinct aspects of the scale-sensitivity of the visual system, and they are modelled compactly:

(1) $$t\left(f,\;E\right)=t_{0}\;\text{exp}\;\left(\left(\frac{f}{f_{0}}\right)\left(\frac{E+E_{2}}{E_{2}}\right)\right)L(f,\;E)$$

This expression is in terms of threshold contrast (*t*). Here, the exponential term captures the high-frequency limb of the sensitivity function, while *L*(*f, e*) captures the low-frequency plateau, which I take to be independent of eccentricity (vi.):

(2) Lf, E=1+α1+ff12

**Figure 1.**
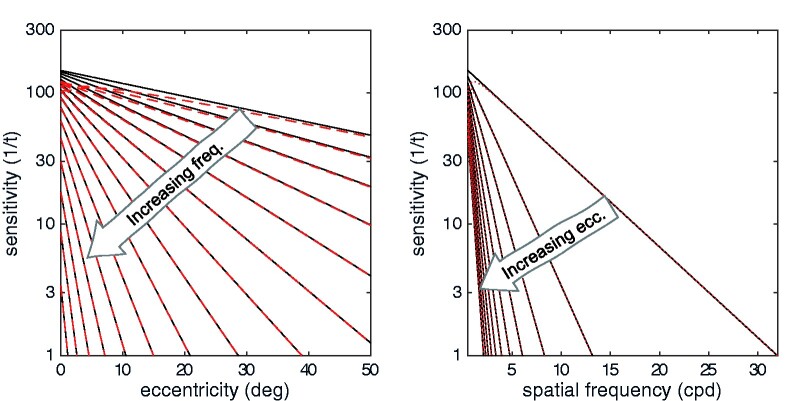
Two overarching properties of human contrast sensitivity. Contrast sensitivity declines with retinal eccentricity and with decreasing scale. Black lines show the exponential relation of contrast sensitivity to eccentricity (left) and spatial frequency (right). An increase in either parameter corresponds to decreased sensitivity. Dashed red lines show a more accurate model with a low-frequency plateau—only the lowest spatial frequencies are meaningfully different.

Regarding the high-frequency component of the function, notice how the exponential declines of sensitivity with eccentricity (E) and spatial frequency (f) are intertwined. There are two constants: *f*_0_ captures the steepness of the decline in sensitivity with increasing spatial frequency (decreasing scale) and *E*_2_ captures the steepness of the decline in sensitivity with increasing eccentricity. In the literature, these two parameters are normally considered separately (see Watson 2018 for a recent synthesis), but it is clear that they modify each other: if *f*_0_ is considered as constant, then *E*_2_ is effectively reduced as frequency increases: the decline in sensitivity for some target (*f*) with eccentricity (*E*) becomes steeper for higher frequencies (Fig. 1A). Conversely, if *E*_2_ is considered as constant, then *f*_0_ is effectively reduced as eccentricity (E) increases, meaning that the decline in threshold with frequency gets steeper (Fig. 1B).

**Figure 2.**
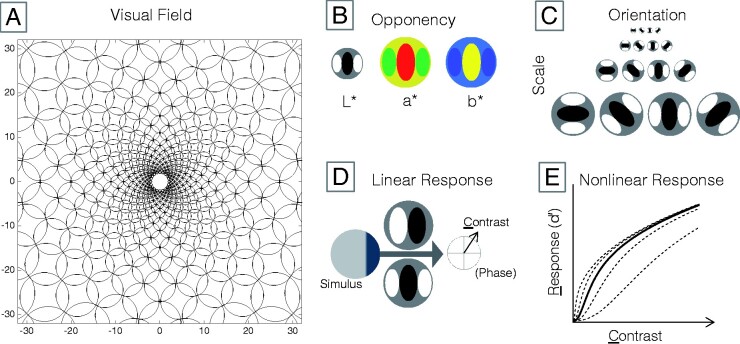
Model of contrast perception. (A) At every point in the model visual field, there is a complement of filters. The scale of the complement only increases with eccentricity. (B) Each filter complement is divided into three colour-opponent channels. (C) Each colour-opponent channel is composed of filters tuned to each combination of four scales and four orientations. (D) Exposed to a stimulus, each filter yields a complex output consisting of a contrast magnitude (C) and its phase. (E) Contrast magnitude is transduced to a signal-noise ratio (d’). The nonlinearity depends on filter scale and eccentricity (Equations 7 and 8), and on local contrast at other orientations (Equation 9).

This connection between the contrast sensitivity functions for spatial frequency and eccentricity has an interesting consequence for sensitivity across the visual field: it means that sensitivity to some frequency *F*_1_ at some eccentricity *E*_1_ is usually equal to sensitivity to a lower frequency *F*_2_ at a larger eccentricity *E*_2_, and to a higher frequency *F*_0_ at some smaller eccentricity *E*_0_. The extreme case of this is the acuity frequency: sensitivity to this frequency is defined everywhere across the visual field as a constant value (*t* = 1). There are, likewise, mechanisms across the visual field with *t* = ½, *t* = ¼, and so-on, with the rule breaking down only at some very low (unknown) contrast.

### Opponent colour channels

The three colour channels were implemented by transforming RGB input images into the CIElab colour space, composed of a luminance-contrast channel (Achr), a blue-yellow channel (B/Y)^1^, and a red-green channel (R/G)—and using these as inputs to the filter layer. The CIElab components are different from the ‘cone contrast’ components typically employed in colour psychophysics, but they are close enough for present purposes (McDermott and Webster 2012). CIElab is also widely available in different code bases and is clearly documented in many sources, making the present results more easily replicable.

### Filter structure

The model is framed in a rectangular array of visual field locations, with each location a 2d coordinate in degrees eccentricity from a point of fixation. At each location a complement of filters is assigned: filters tuned to each combination several spatial scales, four orientations, and three colour contrasts. The filters are created on a log-frequency/orientation domain (Equation 3). The frequency profile is a Gaussian (Equation 4), and the orientation profile is a raised cosine (Equation 5):

(3) $$g\left(f,\theta \right)=g\left(f\right)\cdot g\left(\theta \right)$$

(4) $$g\left(f\right)=\exp\left(-\frac{{\left(\log_{2}f-\log_{2}f_{\mathrm{peak}}\right)}^{2}}{2{\sigma_{f}}^{2}}\right)$$

(5) $$g\left(\theta \right)=\left\{\begin{array}{cc} \frac{1}{2}\left(\cos\left[\sigma_{\theta }\left(\theta -\theta_{\mathrm{peak}}\right)\right]+1\right), & if\;\left|\theta -\theta_{\mathrm{peak}}\right|<\sigma_{\theta }\pi \\ 0, & \;if\;\left|\theta -\theta_{\mathrm{peak}}\right|\geq \sigma_{\theta }\pi \end{array}\right.$$

Frequency bandwidths were fixed at 2*σ*_f_ =1.4 octaves for all filters (Wilson *et al.* 1983). Orientation bandwidth was fixed at 45 degrees (*σ*_θ_ = ¼π). In the space domain, these are quadrature filters, so they encode both amplitude and phase of content in their pass band. The amplitude of each filter was adjusted so that the linear response (dot-product) of the filter to a full-contrast sinewave grating pattern at its preferred orientation and frequency would be unity (1.0), that is the linear response of each filter was explicitly equated with the ‘contrast energy’ (the phase-invariant Michelson contrast magnitude) of a typical contrast sensitivity test stimulus.

The peak orientations of the filters, at every visual field location and for each colour channel, were [0, 45, 90, 135] from vertical. The peak frequencies depended on eccentricity and colour channel. First, the contrast sensitivity function of Equation (1) was fit to data sets for human sensitivity to achromatic (Pointer and Hess 1989), red-green (Anderson *et al.* 1991), and blue-yellow (Mullen and Kingdom 2002) contrast stimuli of variable spatial frequency and eccentricity. Next, the ‘local scale’ of the filters (Watson 1987) was set according to the objective acuity at that eccentricity. The acuity frequency for each colour channel as a function of eccentricity was derived by setting threshold *t*(*f, E*) = 1 and solving for spatial frequency *f*. The peak frequencies (*f*_peak_) of the filters for each channel at each eccentricity were set to:

(6) $$\left\{f_{\mathrm{peak}}\left(E\right)\right\}=\frac{f\left(t=1,E\right)}{\left\{3,6,12,24\right\}}$$

That is, the peak frequency of the finest filter at each location was 1/3 of the acuity limit expected by the pattern of contrast sensitivity, and the coarser filters were spaced 1, 2, and 3 octaves below the finest filter. The low-pass residual from these filters was then inserted to capture the low-frequency plateau of the sensitivity function. This close packing of the frequency bands allowed the filter complement to scale flexibly with eccentricity, avoiding most cases where the coarsest filters might be too large for the test image. For the chromatic filters, three rather than four bandpass filters scales were used along with the low-pass residual.

### Suprathreshold sensitivity

The aspects of contrast sensitivity relating to suprathreshold contrasts (vii, viii and x), and the capacity of the model to fit arbitrary threshold levels (i.e. the shape of the psychometric function for contrast detection or discrimination) are all captured with the Foley transducer (Stromeyer and Klein 1974; Legge and Foley 1980; Foley 1994). This transducer defines the signal-noise ratio (d’) as a nonlinear function of contrast, with parameters that depend on the linear filter characteristics:

(7) $${d\prime }_{\theta }=R_{\max}\frac{{\left({gc}_{\theta }\right)}^{p+q}}{z^{p}+\sum_{\theta }{\left(gc\right)}^{p}}$$

The variable c is the linear response of a spatial filter to the stimulus. In the current study, *p*, *q*, and *R*_max_ are fixed. The threshold parameter *z* varies with eccentricity, frequency and colour. The linear gain g of each filter is a partial parameter that ordinarily would be at unity and distributed between *R*_max_ and *z* (Haun 2009); it was considered separately here as it can account in a computationally convenient way for significant differences in sensitivity between colour channels, allowing other parameters to be fixed. The summation in the denominator is over other orientations of same-location, same-frequency, same-colour filters, thus capturing cross-orientation masking (ix). The threshold for each filter is set by transforming the detection threshold functions defined in Equations 1 and 2, assuming a fixed experimental *d*’ value (*d*’=2 was used in all simulations in this study):

(8) $$z^{p}=\frac{R_{\max}}{{d'}_{\theta }}{t_{\theta }}^{p+q}-{t_{\theta }}^{p}$$

Equation 8 is just a rearrangement of Equation 7, ignoring the cross-orientation terms (basic contrast sensitivity functions are established without masks) and setting contrast equal to an empirical threshold value.

### Fitting model to data

First, data were subjected to a curve-fitting procedure to set the free parameters of the CSF model (Equation 7), with starting parameters suggested by the papers where the model components were introduced (Yang *et al.* 1995; Geisler and Perry 1998; Watson and Ahumada 2005). The nonlinear contrast response parameters (*R*_max_, *p*, *q*) were fixed at the outset to standard values (Legge and Foley 1980; Haun and Peli 2013b). The target data were drawn from the studies listed in Table 1. I did not fit B/Y contrast sensitivity data for the final model, and instead scaled the high-s.f. decline constant (*f*_0_) of the achromatic sensitivity function (see Table 2), and decreased the linear gain, to obtain a good fit to the B/Y acuity data.

**Table 1.** Data sets shown in Figure 3, used to fix the parameters of the contrast sensitivity model.

Data type	Source	
Achromatic contrast sensitivity by spatial frequency and eccentricity	(Pointer and Hess 1989)	Figs. 2, 3, and 12
Red/Green contrast sensitivity by spatial frequency and eccentricity	(Mullen 1991)	Figs. 3, 4
Achromatic and Red/Green acuity by eccentricity	(Anderson *et al.* 1991)	Fig. 5
Achromatic and Blue/Yellow acuity by eccentricity	(Anderson *et al.* 2002)	Fig. 3

**Table 2.** Parameters of the contrast sensitivity model.

	Value		Description
*t*_0_	Achr	0.0051	Overall sensitivity function amplitude (adjusted by g)
	B/Y	0.0051	
	R/G	0.0082	
*E*_2_	Achr	6.22	Eccentricity-scaling constant
	B/Y	6.22	
	R/G	1.82	
α	Achr	5.26	Low-s.f. threshold weight
	B/Y	5.26	
	R/G	2.53	
*f*_0_	Achr	4.51	High-s.f. decline constants
	B/Y	4.51/6	
	R/G	5.37	
*f*_1_	Achr	0.32	Low-s.f. decline constant
	B/Y	0.32	
	R/G	0.94	
*g*	Achr	2.5	Linear (filter) gain
	B/Y	1	
	R/G	2	
*R*_max_	30		Nonlinear gain
*p*	2		Low-c response exponent
*q*	0.4		High-c response exponent

The model was tested by exposing it to actual stimulus images. Each individual filter was convolved with a stimulus, yielding a set of linear measures of contrast at each image location. These linear measures were fed into the response nonlinearity. Responses across scale were combined with a high p-norm ${\bar{R}=\left(\sum_{f}{R(f)}^{M}\right)}^{1/M}$, with *M* = 4; (Cannon and Fullenkamp 1991; Haun and Peli 2013b). The maximum response across the remaining filter dimensions (orientation and colour) is the ‘cross-frequency response’ at each location, and it represents the signal-noise ratio the observer has for making decisions in a SDT task. I constructed achromatic and colour Gabor patch stimuli of varying spatial frequency and centre eccentricity, to (roughly) match the parameters of the exemplar experiments (of Table 1). The contrast of each stimulus was adjusted iteratively (Newton’s method) to produce a peak cross-frequency response equal to the ‘experimental’ d’=2 (an unbiased yes/no hit-rate of 84%)—this contrast was the model’s detection threshold for the target stimulus.

**Figure 3.**
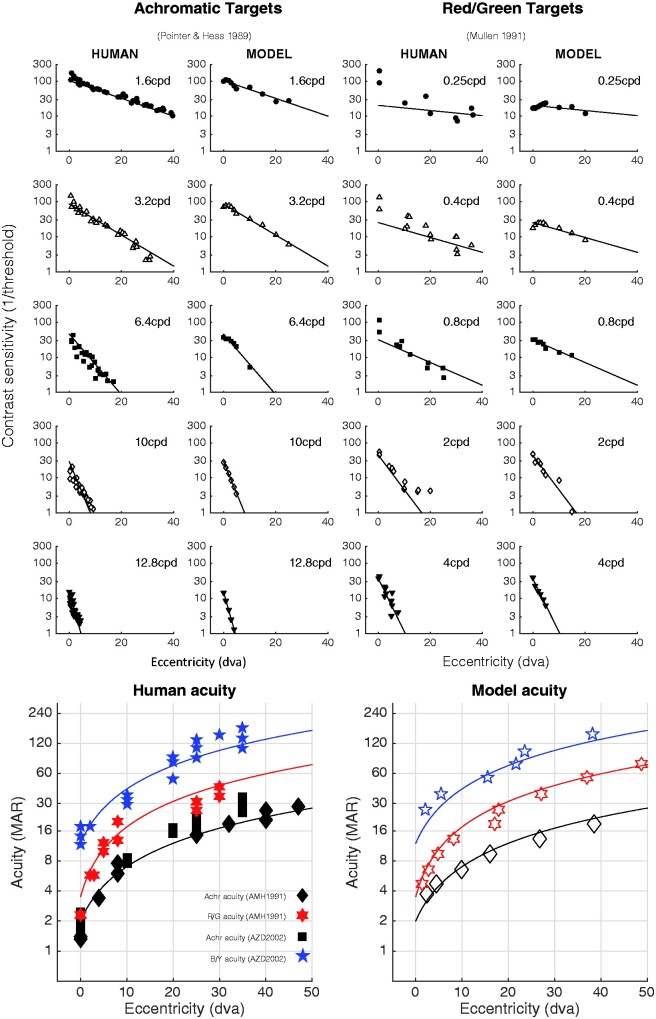
The contrast sensitivity and acuity of the model are similar to that of human observers. The upper panels show human and model contrast sensitivity estimates for achromatic (left) and red/green (right) targets, for different target spatial frequencies (cycles per degree, cpd) and retinal eccentricities (in degrees of visual angle, dva). The straight lines are regression fits to the log-CS human data, and are the same in both data and model plots, to aid in by-eye comparison. The lower panels show acuity for achromatic, blue/yellow, and red/green grating stimuli; solid symbols are human acuity, open symbols are model acuity. The solid lines are linear fits to the human data, and are the same in both human and model panels.

Model thresholds are plotted in Fig. 3 against data from Mullen (1991) and Pointer and Hess (1989). Also shown are acuity estimates—these are obtained by extrapolating the contrast sensitivity curves to the spatial frequency where sensitivity = 1. In addition to these basic limits of contrast detection, the model also captures suprathreshold aspects of contrast sensitivity. Contrast discrimination (threshold-vs-contrast) functions have the familiar ‘dipper’ shape, where increment thresholds for just-detectable pedestals are smaller than the detection threshold itself, while increment thresholds for suprathreshold pedestals follow a compressive power function (Stevens’ law). Cross-oriented masks elevate the dipper functions in the expected way, though they are much more powerful masks than they should be (since the cross-oriented inputs were not realistically weighted).

In summary, the model generally comes very close to human performance. Whatever conclusions one might draw from the empirical data should transfer well to the simulated data. That is, if one believes (for example) that human colour or sharpness representations must decline in quality due to the observed pattern of contrast sensitivity, then the same belief should apply to the representations of this model. Put another way, the facts pertinent to claims about what a human observer can and cannot experience at the level of contrast perception are all effectively embodied by this model.

### Application of the model to natural scenes

**Figure 4.**
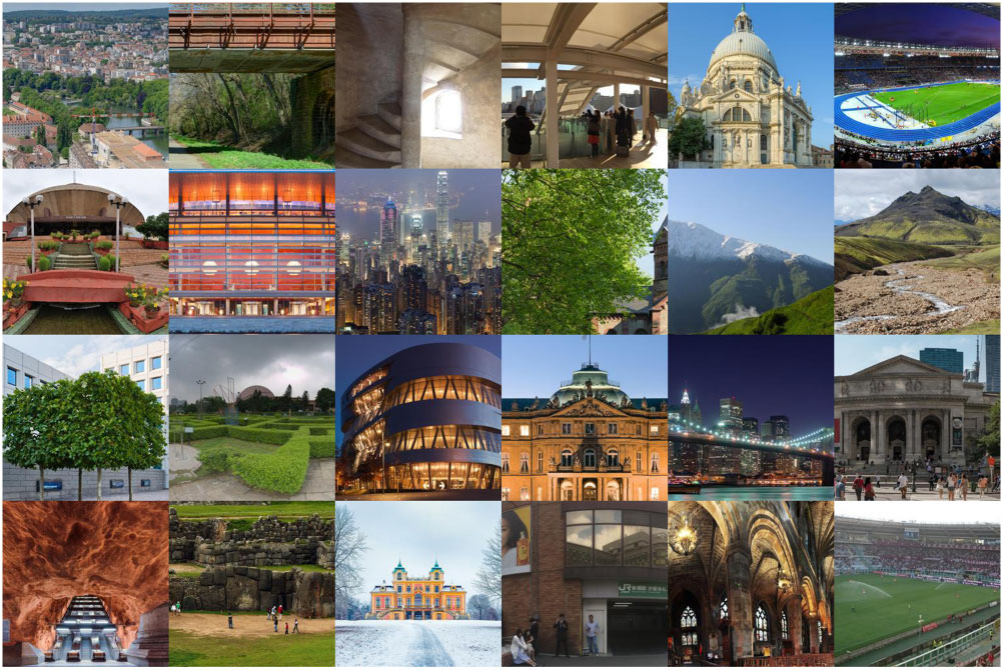
Some of the images used as stimuli for the model.

The central question in this study can be put this way: given what we know about human contrast sensitivity, what can a typical human observer see in a colourful natural scene? To answer this question I used 100 colourful natural scene photos (Fig. 4) as stimuli for the model. The scenes were all pulled from the ‘Panorama’ section of the Wikipedia directory of featured pictures (Various, 2019); these images are very high resolution, minimally compressed, and full-colour, and they are of the kinds of interesting vistas that might elicit naïve claims about the apparent vividness of a visual experience. The main selection criterion was that each image must have height and width equal to or greater than 1536 pixels. Inputs to the model were cropped to 1536 × 1536 pixels; if an image had least dimension greater than 3072 pixels, it was cropped down to the nearest multiple of 1536 and then resized down to 1536 × 1536. Some subjective criteria were applied in selecting the scenes, including that the central region of the scene should contain some more ‘interesting’ content than just ground (or sea) and/or sky; some content should be ‘near’, that is obviously telescopic images were excluded; images should seem colourful (scenes like pictures of the desert or of snowy mountains that seemed effectively monochromatic were generally not included, though some were); and a rough balance was sought between ‘natural’ and ‘artificial’ scenes (i.e. of scenes with and without obvious human influence). The list of source images (URLs and photographer credits) is provided in the Appendix. I did not try to match the ‘true’ visual angle of the scenes to the visual angle of the model’s visual field—the necessarily information to recover the true angle was not generally included with the images.

**Figure 5.**
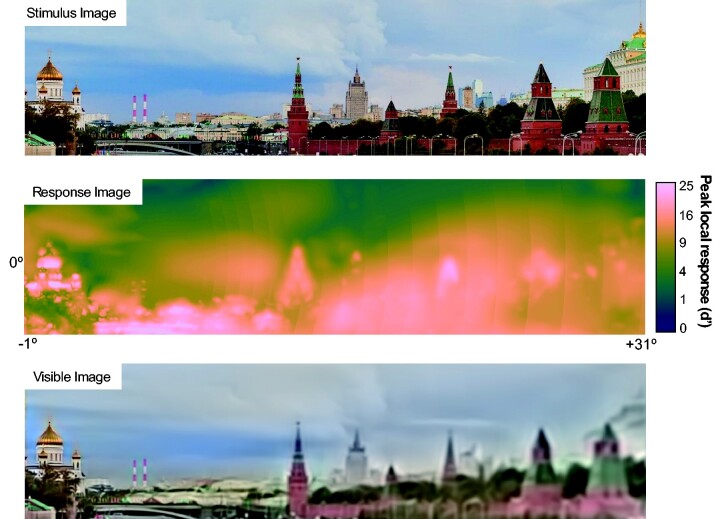
Top: Stimulus image exposed to the model. Middle: Model response image to the stimulus, collapsed across scale, orientation, and colour (using the M-norm for scale and winner-take-all for orientation and colour). Notice that most of the scene is taken up by very high d’ values (shades of bright yellow): At these locations, content is easily visible. The colormap is batlow from Crameri (2020). Bottom: The ‘visible image’ according to the model response. This image is composed only of image contrasts that elicit ‘suprathreshold’ responses.

To ‘view’ the scenes, the model was given a 32° square visual field (which would fit comfortably within the angle of view of photographs not taken with wide-angle or long-focus lenses), extending from 1° left of the fixation point to 31° right; and from 16° below to 16° above. The resolution of the field was 1536 × 1536 pixels. The model’s response (in the form of the maximum cross-frequency response at each field location) to one stimulus image is shown in Fig. 5. Right away we learn something about natural scenes: they are composed of high contrasts, as far as the visual system is concerned. Detection thresholds are routinely exceeded across the model visual field, as shown by the very-high *d*’ values elicited across the scene. If we were doing signal detection experiments with the image components evoking these responses, an observer would respond perfectly (barring finger errors or attentional lapses) across thousands of trials.

## Results

Our original questions concern whether the content of natural scene experiences is rightly characterized as ‘colourful’ and ‘sharp’ across the extent of the visual field. So, how do we evaluate these qualities? Colourfulness is the more straightforward to address, so we start there.

### Colourfulness

‘Colourfulness’ is an informal term, but in its informal usage it is intended to imply either variegation (i.e. many distinct hues) or vividness (i.e. the presence of a highly saturated colour) of colours, or especially both. Here, I used the ‘hue-saturation-value’ (HSV) representation of colour content to capture these features (Smith 1978).

*The ‘visible image’.* HSV is a pixelwise representation of image content, whereas the spatial vision model generates a high-dimensional matrix of signal-noise ratios. However, it is straightforward to translate the model’s response to an image back into the form of a ‘visible image’, where we can make use of HSV. To do this, we transform each filter’s signal-noise ratio (*d*’) into the range [0,1], using this value to weight the positive cosine phase of the filter, and adding all the weighted filters to an output image. The appropriate transformation of *d*’ is the ‘accuracy’ or ‘reliability’: the greatest difference between the yes/no hit-rate and false-alarm rate:

(9) $$\mathrm{Accuracy}=HR-FA=\Phi \left(\frac{R}{2}\right)-\Phi \left(-\frac{R}{2}\right)$$

Here, Φ is the cumulative normal distribution function, *R* is the *d*’ elicited by the stimulus. This procedure maps near-zero *d*’ to near-zero accuracy, and higher *d*’ to accuracy approaching one. The *visible image* is then composed of content only to the degree that it elicits psychophysically accurate responses. That is, if a filter response is highly likely to have been elicited by a stimulus (a hit), and highly unlikely to have been due to intrinsic noise (a false alarm), then its contrast is considered ‘visible’ and composes a part of the output image.

The reconstructed image is in CIElab coordinates, and is transformed to HSV coordinates by chaining the MATLAB lab2rgb and rgb2hsv functions.

*Sampling across the visual field.* An example of a ‘visible image’ is shown at the bottom of Fig. 5. The image seems similar to the original, except for its reduced peripheral resolution. Its colourfulness does not *seem* reduced across the model field’s extent, but the eye is the wrong judge here (the ‘double-pass problem’: cf. Peli 1996). This is where we make use of the HSV representation, and sample hue variation and saturation across the field. What is the right way to do this? A human observer making a judgment about some image property across the visual field must be using spatial attention, and it is known that the size of the spatial attention ‘spotlight’ varies with eccentricity, with a radius *r* following a pattern much like the scaling of acuity (Intriligator and Cavanagh 2001):

(10) r=k01+EE2+kc

Here, I followed Intriligator and Cavanaugh’s results (Intriligator and Cavanagh 2001) and set k_0_ to 5 minutes of arc, and *E*_2_ to 0.34 degrees eccentricity; but since this results in foveal windows just a few pixels across (which would result in severe undersampling at the fovea of local quality values for the measures described below) a constant *k_c_* of 1 degree was added at all eccentricities. This eccentricity-scaled sampling rule reflects the attentional constraints on the spatial sampling strategy of a human observer tasked with investigating the local spatial distributions of some visual quality like colour.

One could argue that the scaled sampling rule is biased with respect to the central research question, so the scaled rule was compared with an unscaled sampling rule, fixing r at 3.75 degrees. This is the median (from 0 to 31 degrees eccentricity) of the scaled sample window, and is about the size of the parafoveal region. (It is also the radius of the round window made by touching the tips of my thumb and forefinger at a viewing distance of about 40 cm).

**Figure 6.**
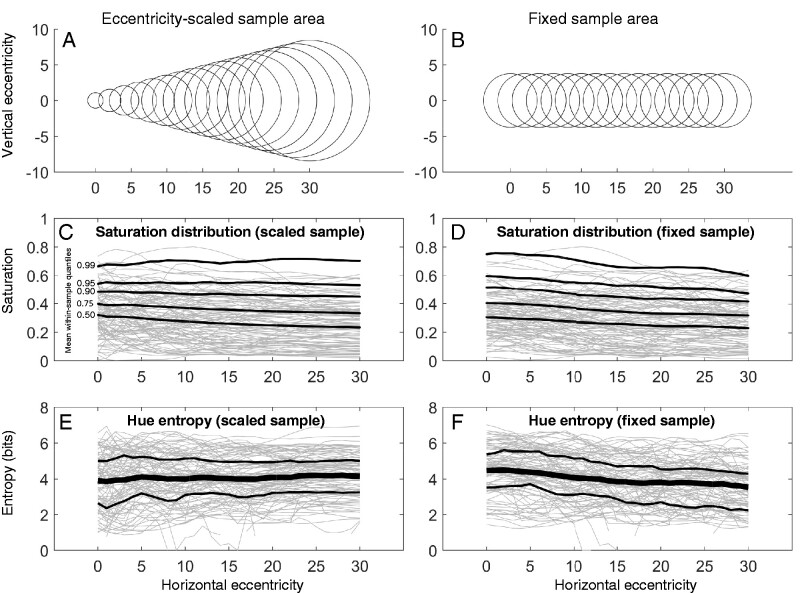
Colourfulness statistics as a function of eccentricity, for eccentricity-scaled (A) and fixed (B) sample areas. (C, D) Saturation distributions. Each grey line is the mean HSV saturation, over eccentricity, for one image. The black lines trace the average upper quantiles (0.5, 0.75, 0.9, 0.95, and 0.99) over all images. (E, F) Hue entropy. Each grey line is the average hue entropy, over eccentricity, for one image. The black lines trace the average entropy over all images. The vertical bars are upper and lower entropy quartiles (0.25, 0.75) over all images.

*Colourfulness over eccentricity.* With these sampling rules, we assess colourfulness of model-visible images at each position (in 1-degree steps) along the horizontal midline as shown in Fig. 6. Two measures capture colourfulness at any sample position: first, there is the distribution of saturations, with high saturation quantiles reflecting the most colourful parts of the sample; second, there is the hue entropy, which reflects the variegation of a sample (how many different hues are encountered there). Intuitively, the hue entropy should be computed for saturations that produce visible colours—for this demonstration a relatively low bar of 0.2 saturation was set, since a higher bar tended to reduce sample sizes to zero for many scenes, even near the fovea (very saturated colours in natural images are relatively rare; Long *et al.* 2006). Saturation quantiles are self-explanatory, and hue entropy (H) is defined as:

(11) $$H=-\sum_{\mathrm{hues}}p\left(\mathrm{hue}|\mathrm{sat}>0.2\right)\;\log_{2}p\left(\mathrm{hue}|\mathrm{sat}>0.2\right)$$

The distribution *p(hue | sat>lim)* was defined over 256 HSV hue bins. Maximum entropy—an even distribution of hues across the full range—would be H = 8 bits. Concentration of colour around particular hues appears as lower entropy.

Fig. 6C and D shows the upper quantiles of the saturation distribution as a function of eccentricity for the different sampling rules; Fig. 6E and F shows the hue entropy. For the scaled rule, there is little dependence on eccentricity of the saturation distribution. For the fixed rule, there is a gradual decline with eccentricity in the saturation of the highest quantiles. The relationship between eccentricity and hue entropy is similarly muted: entropy increases slightly with eccentricity for the scaled rule (for the scaled rule the average increase in entropy is about 0.01 bits per degree), and decreases slightly for the fixed rule (on average losing about 0.04 bits per degree). Entropy is generally around 4 bits in the investigated range.

Considering that the fixed-size sampling rule is unrealistic and probably perceptually impossible, the slight declines in colourfulness for that rule are not what we should expect a human observer to report. At the same time, despite its relative ‘flattening’ by adding 1 degree of radius at each eccentricity, the attention-scaled rule might impose too-rigid a frame to visual field sampling: perhaps observers (especially expert observers) are, with some effort, able to attend much smaller zones in the periphery and much larger zones nearer the fovea, when they are trying to ‘sample fairly’ across the visual field. So the scaled rule might likewise overestimate the relationship in the opposite direction. Left with a relationship somewhere in between a slight increase and a slight decrease, it seems reasonable to describe the relationship between visible colourfulness and retinal eccentricity as negligible.

### Sharpness

There is no standard pixelwise measure of ‘sharpness’ analogous to hue and saturation. What could be the response image correlate of apparent sharpness? It is useful to define sharpness negatively, as the absence of apparent blur: if a feature is seen but does not appear blurry, then it appears sharp. The spatial spread of ‘just detectable blur’ increases in proportion to eccentricity in a similar way as acuity (Hess *et al.* 1989; Levi and Klein 1990; Wang and Ciuffreda 2005; Maiello *et al.* 2017). That is, across the visual field, if the spatial spread of blur is less than the acuity limit, a ‘blur percept’ will not be evoked; but if the spread is larger, it will be. So, a simple model of sharpness should capture whether or not content at some position in the visual field (especially ‘feature’ content) extends all the way to the acuity limit.

**Figure 7.**
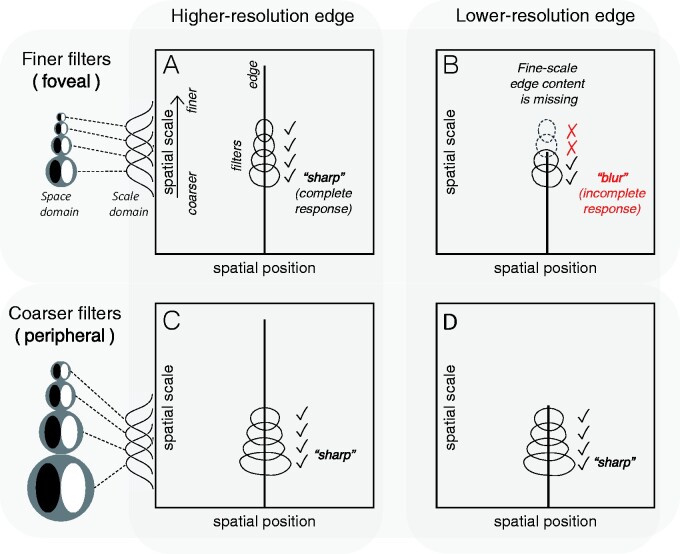
The ‘scale space’ model of edge sharpness and blur. Two kinds of physical stimulus are illustrated: a high-resolution (physically sharp) feature at left, and a low-resolution (physically blurred) feature at right. These are represented as lines indicating existence of content across a range of scales, at a single spatial position: the high-resolution edge has content across a broad range of scales (A, C), while the low-resolution edge is missing the fine-scale content (B, D). The high-resolution edge evokes a *complete response* in a set of fine-scale filters like those found in the fovea (A), corresponding to perceptual *sharpness*, while the low-resolution edge evokes an *incomplete response* (B)—some of the filters do not respond—corresponding to perceptual *blurriness*. However, both features evoke complete responses for a set of coarser-scale filters like those found in the periphery (C, D).

A measure applicable to the multiscale channel contrast responses of our model can be derived from the ‘scale space’ notion of feature representation (Koenderink 1984; Witkin 1987; Perona and Malik 1990; Georgeson *et al.* 2007). Fig. 7 uses this concept to illustrate the distinction between physical sharpness (e.g. ‘high resolution’) and perceived sharpness (‘perceptual clarity’), and to explain how we can find a correlate of apparent sharpness in the spatial vision model. These properties (physical and perceived sharpness) may often be conflated, but they are just as distinct as, for example, reflectance spectrum and perceived colour. The left two panels (A, C) show the scale-space representation of a high-resolution edge: such an edge exists, in physical terms, as a feature at a particular spatial region (on the *x*-axis) extending from coarse scales up to fine scales. The right panels (B, D) show a low-resolution edge: this edge exists as a feature that extends from coarse scales up to only moderately fine scales. These are two physically different features, but they do not determine perceptual qualities: perceptual mechanisms also have to be taken into account. The upper and lower panels contrast two different filter scales—a fine-scale ‘foveal’ filter set, and a coarse-scale ‘peripheral’ set. The foveal filters distinguish between the two features in that the lower-resolution edge does not elicit any response from the smallest filters (Fig. 7B). So, the high-resolution edge elicits a ‘complete’ filter response, and the low-resolution edge elicits an ‘incomplete’ response. The peripheral filters do not distinguish the two features: for this filter set, both edges elicit ‘complete’ responses.

The implication of the scale-space demonstration is that ‘apparent sharpness’ is closely related to a complete filter response, and is therefore distinct from the physical resolution of a stimulus. This link between apparent sharpness (and blur) and a cross-scale response has been proposed many times, though always in different forms (e.g. Elder and Zucker 1996; Wang and Simoncelli 2003; Georgeson *et al.* 2007), and usually in reference to foveal perception (one exception is Anstis 1998). Going by this way of thinking about apparent sharpness, we can recruit the ‘response accuracy’ statistic of Equation (9) and define apparent sharpness as the cross-scale product of accuracies at a given location:

(12) $${FC}_{\theta }=\prod_{f}\mathrm{Accuracy}\left(R_{f,\theta }\right)$$

This ‘filter completeness’ measure (FC), approaches a value of 1 when all similarly oriented filters at some spatial position are responding strongly, as would happen in the presence of an oriented feature that is at least as finely resolved as the smallest filter.

**Figure 8.**
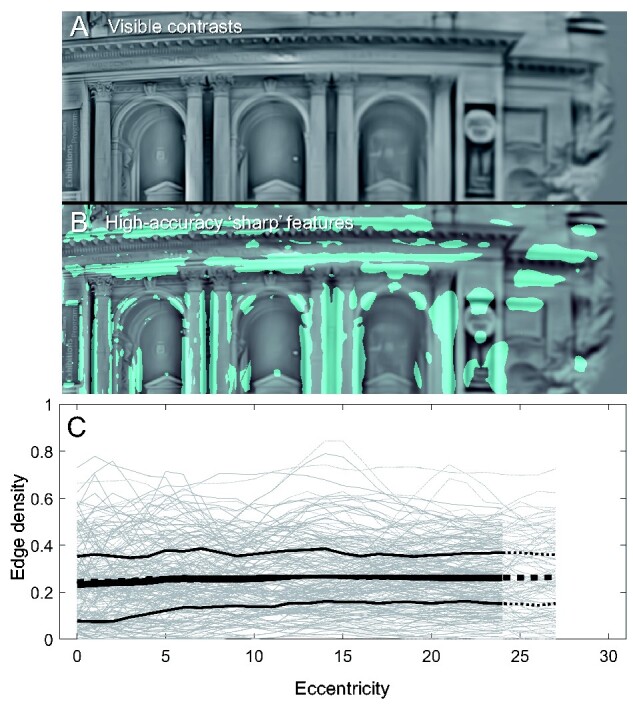
(A) The visible image of a scene’s luminance contrast. (B) The regions of the scene where responses are filter complete (*FC*_θ_ >0.96) are coloured cyan. Note how ‘sharp’ features are themselves progressively larger in angular size, with increasing eccentricity (from left to right). (C) The proportion of *FC*_θ_ >0.96 pixels (‘Edge density’) within the sampling window positioned at each horizontal eccentricity. Grey lines are measures for each scene (*N* = 100), black lines are the mean and upper/lower quartiles over all scenes for the scaled sampling rule. Dotted lines are for the fixed sampling rule (they do not differ appreciably from the scaled rule). The average was not taken for windows extending outside the model field.

Fig. 8 illustrates the application of this idea to the model responses to natural cenes, taking filter completeness to be *FC*_θ_ values greater than 0.96 (allowing that each of four filter responses has accuracy ∼0.99)^2^. Here, I evaluate filter completeness only for the luminance channel, since its smaller filter size means that it must in any case be the driver of sharpness judgments. The first panel (A) shows the visible luminance-contrast image for a particular scene; the next panel (B) highlights the regions of the image where the model response was ‘filter complete’. Using the same two sampling rules as in the colourfulness analysis, the last panel (C) shows the average filter completeness—the mean of *FC*_θ_ >0.96 within the sample region—as a function of (horizontal) eccentricity. This analysis is insensitive to sampling rule, but there is a clear positive trend with eccentricity of increasing filter completeness. Under the hypothesis that filter completeness underlies apparent sharpness, the spatial vision model does not support the notion that apparent sharpness should decline with eccentricity.

**Figure 9.**
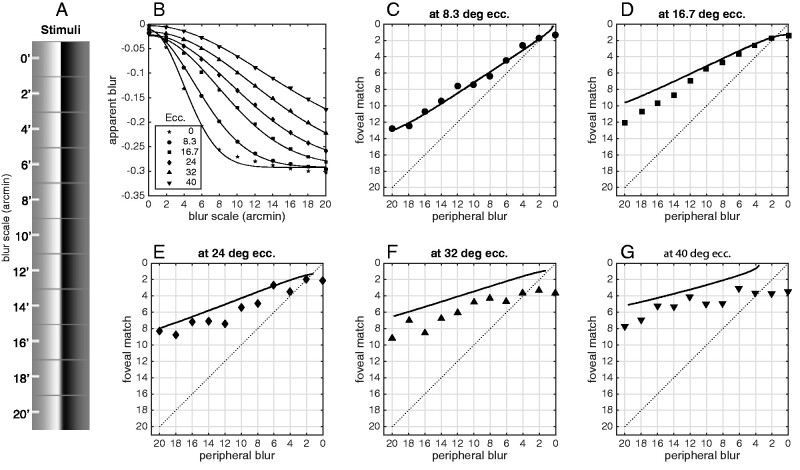
(A) Stimuli used for the replication of Galvin *et al* (1997). Eleven levels of gaussian blur were applied to a monochrome vertical edge. The stimuli are illustrated to scale: the image is 10.67 degrees wide. (B) The ‘apparent blur’ statistic β for a simple edge stimulus convolved with a gaussian with the scale constant on the *x*-axis (scale constant is in minutes of arc, i.e. 1°/60). The apparent blur metric is explained in the text. Each line is a Gaussian fit to the blur metric as a function of stimulus blur, for each of six stimulus eccentricities. (C-G) Apparent blur matched between a fixed-blur peripheral stimulus and an adjustable-blur foveal stimulus. Model matches between the foveal and peripheral blurs were computed numerically using the gaussian curves fitted in (B). Veridical matches would be on the main diagonal, and matches above the main diagonal mean that the peripheral stimulus appears sharper (less blurry) than it would if it were viewed foveally. Symbols are data from Galvin *et al* (1997)’s first experiment (replotted from their Figure 2). As eccentricity increases, the model becomes less accurate, over-estimating perceived sharpness of peripheral content. The model judges peripheral edges to be even sharper than the human observers judged them to be. Over the five test eccentricities {8.3, 16.7, 24, 32, ana 40} the average difference between model and data is small but consistently negative: {0.2, −1.0, −1.1, −2.0, and −1.9} arcmin, respectively.

*Apparent blur of visible content.* Apparent sharpness does not capture the whole story: if a feature does not appear sharp, then it must appear blurry, and there is plenty of room for features to appear more or less blurry depending on various circumstances. Apparent blur, that is how blurry something appears to be (with ‘sharpness’ being the minimum of apparent blur), is usually measured by perceptually matching the blur of one percept to the blur of another. In a pair of studies especially relevant to the central question of this study, Galvin *et al.* (1997, 1999) measured apparent blur matches between foveal and peripheral stimulus positions. They found that a blurred edge viewed peripherally was subjectively matched to a less-blurred edge viewed foveally (scattered symbols in Fig. 9C–G). They called this effect ‘sharpness over-constancy’. They proposed that some mechanism corrects for the lower resolution of peripheral vision. In their view, peripheral stimuli appear *sharper than they should*: implicitly they were taking ‘foveal appearance’ as the standard for how things ought-to-look. The spatial vision model suggests a different interpretation of their results. Fig. 9 replots data from Galvin *et al’*s Experiment 1, along with perceived-blur matching functions from the spatial vision model. ‘Apparent blur’ does not have an easy implementation in the scale space model, so I adapted the simple ‘response slope’ model of Elliott *et al.* (2011) and Haun and Peli (2013a). In their model, apparent blur is equated to the rate of decrease (*m*) of the perceptual response (here *R*) as the log filter scale decreases (as log center frequency *f* increases):

(12) $$R\left(f\right)=m\ln\left(f\right)+b$$

This model was originally designed to explain perceived blur of a special class of stimuli (blurred by steepening the amplitude spectrum slope; Elliott *et al.* 2011; Haun and Peli 2013a), and it performs very badly (i.e. non-monotonically for increasing blur levels) for gaussian-blurred stimuli. However, I found that adjusting the slope term by the local response (the *M* = 4 norm $\bar{R}$) yields blur estimates that monotonically increase with stimulus blur (Fig. 6a), so the ‘apparent blur’ term β is:

(13) $$\beta =\frac{m}{\bar{R}}$$

When there is similar response across filter scale (as would be ideally evoked by the large edge stimuli of the Galvin *et al.* study), β will be near zero—when response declines with increasing filter scale, β will be negative. β can also run to positive values (‘oversharpness’) when there is relatively more fine-scale than coarse-scale content, as with a fine-grained texture.

This perceived-blur model (as with most or all others proposed) has been tested only with foveal psychophysics data, and it fits the matching Galvin data only roughly (Fig. 9C–G). However, notice how it fails: held to a foveal standard, the spatial vision model behaves as though peripheral content, especially at larger eccentricities, is perceived as even less blurry than Galvin *et al.* found it to be (Fig. 9E–G). That is, rather than sharpness over-constancy, there may be an under-constancy at work in human peripheral vision. One possible culprit here is crowding (Rosenholtz 2016): a subject’s relative inability to judge the qualities of content in peripheral vision, despite the psychophysical availability of the necessary information, might contribute to judgments of apparent blur (does a crowded display feel more blurry?). Or, it could be that observers have some natural knowledge that the objective resolution of peripheral vision is less than that of foveal vision, and they are injecting that knowledge into their decisions about apparent peripheral blur. Finally, the model of Equations 12 and 13 might simply be inadequate. At any rate, the spatial vision model does not predict that peripheral stimuli should be judged as blurrier than foveal stimuli, or indeed as blurrier than human observers themselves tend to judge them.

*Apparent blur and sharpness of natural scenes.* The apparent blur model of the previous section is straightforward to apply to the natural scene contrast responses underlying the analyses in previous sections. This analysis requires the obvious caveat that, as shown in Fig. 9, the apparent blur model is a rough fit to the one available data set (Galvin *et al.* 1997). Also, except for the example of Fig. 9, the model has never been validated on local image patches, only on ‘whole image’ statistics (Elliott *et al.* 2011; Haun and Peli 2013a). However, the model is not that far off the Galvin *et al.* results—it closely matches data at smaller eccentricities, and is at least monotonic with the psychophysical patterns.

**Figure 10.**
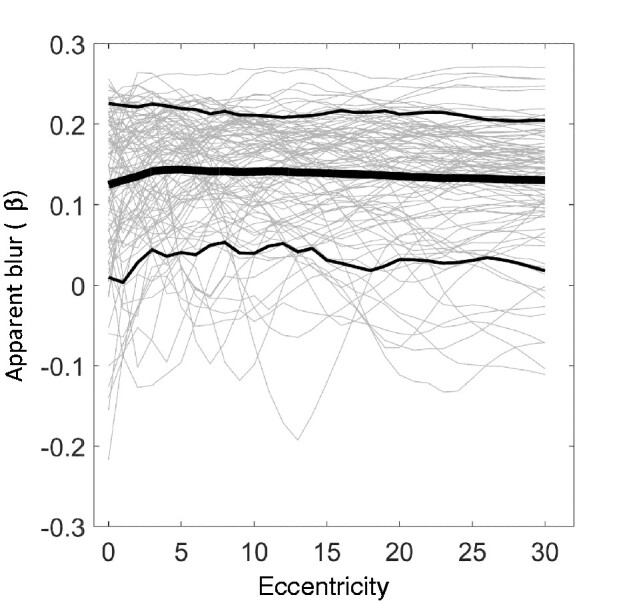
(A) The ‘apparent blur’ measure over eccentricity for the scene stimulus set. Unlike the simple edge stimuli of Figure 6, natural scenes typically evoke positive apparent blur scores, which we may interpret as ‘apparent sharpness’. In general, regions of a natural scene at optical infinity (as in our stimulus set) will evoke these positive scores; negative scores generally correspond to featureless regions, usually sky, where most content is in a very low-frequency gradient.

Fig. 10 shows how the apparent blur parameter β, as evoked by the scene images, varies with eccentricity: it does not vary much at all, averaging a positive value at every eccentricity. If, in viewing a panorama, normal human observers are comparing some statistic like β across their visual fields, they should find that the distribution of apparent blurs is not obviously dependent on retinal position. In fact, if the intrinsic blur statistic is anything like β, they should find that a typical scene (that is, one viewed at optical infinity, as with most of our sample scenes) does not contain much blur at all.

### Attention

The target data on which the model is constructed and tested were all collected under conditions where the stimulus was the sole focus of the observer’s spatial attention. So, the findings would seem to hold for, at a minimum, judgments about attended colour and sharpness qualities. What happens to colourfulness and sharpness when spatial attention is withdrawn from a region of the visual field?

The effects of spatial attention are complex (involving shifting tuning for individual neurons, changes to perceptual organization and recognition, and surprising phenomena like inattentional blindness) and, in general, its mechanisms are poorly understood. However, at the level of early spatial vision, we have some idea of what is happening. Neural and psychophysical measures seem to agree that spatial attention corresponds to enhancement of contrast response (Buracas and Boynton 2007; Herrmann *et al.* 2009; Reynolds and Heeger 2009; Bressler *et al.* 2013; Guo *et al.* 2020); so, withdrawal of spatial attention means reduced contrast sensitivity, reduced perceived contrast (but see Schneider and Komlos 2008), and attenuated neural response. A crude implementation of this enhancement (or of its withdrawal) in the model would be to vary the amplitude (*R*_max_ in Equation 9) of the contrast response function. If the main model reflects the enhanced response state of an attended region, we can implement the withdrawal of attention by reducing *R*_max_. Reducing this parameter would mirror the kinds of reductions seen in neural contrast response functions (Luck *et al.* 1997; Gandhi *et al.* 1999; Buracas and Boynton 2007), and would also reduce contrast sensitivity and perceived contrast judgments (assuming that perceived contrast is strongly linked to contrast response magnitude) in similar ways to what is observed psychophysically (Foley and Schwarz 1998; Carrasco *et al.* 2000, 2004; Huang and Dobkins 2005; Carrasco and Barbot 2019).

**Figure 11.**
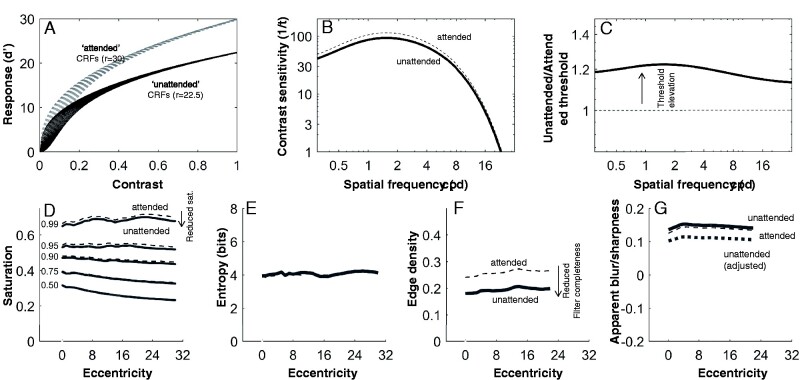
Simulating the effects of attention on visibility of colour and sharpness. (A) Attended (*R*_max_ = 30) and unattended (*R*_max_ = 22.5) contrast response functions for a range of *z*-values (threshold parameters). (B) Unattended (solid line) contrast sensitivity is reduced relative to attended (dashed line) sensitivity. The sensitivity curve here is for foveal luminance contrast before the linear gain parameter (*g*_ach_). (C) Plotted as threshold elevation, the difference between unattended and attended thresholds tends to around 20%. (D) There is some reduction of visible saturation for the unattended condition. (E) Visible hue entropy is not affected by the attention manipulation. (F) Visible edge density is reduced in the unattended condition. (G) Apparent sharpness (positive values of the apparent blur metric) is *increased* by inattention. The thick dotted line shows an adjusted metric that assumes knowledge of the inattentional reduction in response amplitude.

I repeated the colourfulness and sharpness analyses using a version of the model with *R*_max_ reduced by 25% (Fig. 11A, *R*_max_ = 22.5; this reduction is consistent with the magnitude of attentional effect on fMRI BOLD response). This is a significant reduction that produces psychophysical effects in a similar range to what has been observed in numerous studies, reducing sensitivity by around 20% (Fig. 11B and C), depending on the default sensitivity of each mechanism, but I did not try to fit the reduction to any particular data set (see Lee *et al.* 1997 and Carrasco *et al.* 2000 for similar effects; many other effects of similar magnitude are reviewed in Carrasco (2011).

Fig. 11 also compares the original perceptual quality measures of the ‘attended’ scenes with measures of the ‘unattended’ scenes. The changes to colourfulness are modest: unattended regions have reduced saturation (99th percentile saturations, on average, drop from 0.54 to 0.51; Fig. 11D) and slightly reduced hue entropy (from 4.31 to 4.25 bits; Fig. 8E). The changes to sharpness are larger (Fig. 11F): edge density (averaged over eccentricity) drops from around 0.27 to around 0.21, roughly proportional to the change in response amplitude. Interestingly, the apparent blur metric (β) *increases* slightly with inattention (Fig. 11G)—while decreasing *R*_max_ would reduce the slope estimates underlying the blur metric, the normalizing factor, being decreased by the same factor, over-compensates for the reduction. If we think that apparent blur should change similarly to edge density, we can suppose that the visual system ‘knows’ about the inattentional reduction of *R*_max_, and takes this reduction into account by reducing R-based statistics by the same proportion: basically, we multiply the original β by the reduction factor. The dotted line in Fig. 11G shows this adjusted β is similarly reduced to the reduction in edge density.

These attentional effects on visible qualities are not very dramatic, but they are real. If the actual effects of withdrawing attention on contrast sensitivity are larger than what is modelled here, then the effects on perceptual qualities would be correspondingly larger. Overall, this may support a weak version of the so-called ‘refrigerator light illusion’ (Block 2001), which is the notion that unattended properties of visual experience may be somewhat different from attended properties—but that we would not notice the difference, since whenever we check we find the attended versions of those properties.

Why, if the contrast response (and sensitivity) is changed so significantly, are visible qualities not more dramatically affected? The answer is that saturated colours or sharp details are evoked by high physical contrasts that yield (in the attended case) very high signal-noise ratios; halving these ratios will generally still result in a suprathreshold response (e.g. going from *d*’=8 to 4). If halving the contrast response results in a large decline in the accuracy of a feature, then the attended response must already have been rather weak (e.g. from *d*’=2 to 1). In terms of accuracy (Equation 9), the reduction of contrast response magnitude only has meaningful consequences for features whose attended response was in the range [0,∼4]. Responses in this range already contribute only marginally to colourfulness and sharpness (this is almost by-definition: *of course* low contrasts do not contribute much to judgments of sharp edges or vivid colour). The effects of attentional enhancement of contrast response are on the faint and hard-to-see, rather than on the vivid and easy-to-see.

## Discussion

**Figure 12.**
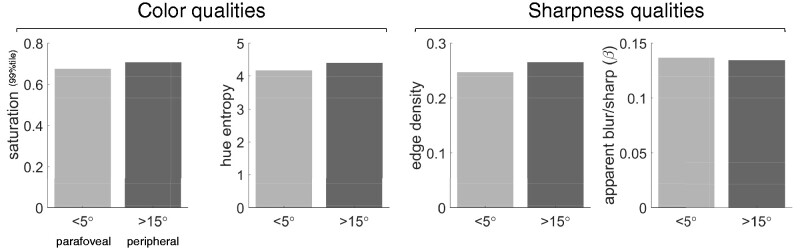
A summary of the main results. Colour qualities and sharpness qualities, as assessed against contrast responses to colourful panoramic scenes, do not differ dramatically between parafoveal and peripheral visual fields. The values here are the arithmetic means of values below 5 degrees (for a ‘parafoveal’ measure) and above 15 degrees (for a ‘peripheral’ measure) for data shown in Figs. 6, 8, and 10.

According to standard techniques for measuring human vision, basic capacities of visual perception (sensitivity and resolution) decline significantly with increasing retinal eccentricity. These facts have led some to conclude that perceptual *qualities* must therefore degrade with eccentricity. To the contrary, however, the present study shows that, given the sensitivity and resolution of the normal human observer, one would expect perceptual qualities to be rather stable across the visual field (Fig. 12). This demonstration requires only that we take an intrinsic perspective on spatial vision: that the visual system can only know about what it can represent; it cannot know about what it cannot represent. This idea was expressed particularly well by Anstis in his 1998 paper on understanding peripheral acuity:

“Why does the whole visual field normally look equally sharp all over, when there is clearly an enormous degradation of the visible detail in peripheral vision? This is an ill-posed question. After all, if our acuity were 1000 times better than it is, we could throw away our microscopes, yet our 'limited’ foveal acuity which prevents us from seeing bacteria with the naked eye never looks like any kind of subjective blur! The same applies to our limited peripheral acuity. A channel cannot signal anything about stimuli to which it is not tuned, so peripheral retinal channels must remain silent in the presence—or absence—of high spatial frequencies to which they are blind.” (Anstis 1998)

This way of understanding visual perception might seem straightforward, but an ‘extrinsic’ perspective on vision may be closer to the mainstream of cognitive science, since it fits well with overarching theories of computation and information-processing, and notions of veridicality and intentionality (some distinct recent critiques of the extrinsic perspective include, Lehky *et al.* 2013; Hoffman and Prakash 2014; Brette 2019). That is, we tend to see perception—and perceptual experience, specifically—as a process involving an external signal, an internal response or representation, and mechanisms linking the two. Under this perspective, it may seem intuitive that, because there are small image features that can be seen foveally but not peripherally, peripheral vision is actually blurry in comparison to foveal vision. The fact that it does not *seem* this way then produces an excitingly counterintuitive thesis: that the visual field does not really feel the way it *seems* to feel, and introspective judgments about visual qualities are not to be trusted. However, perceptual experience is, by most accounts, intrinsic to the observer, and cannot involve the stimuli per se: if there are stimulus properties that are not represented in experience, their absence does not figure in the experience. Rather, the experience is entirely a matter of the structure of the representation. Because the structure of edge representation in the periphery and the fovea is similar, they may well be experienced in very similar ways, despite the fact that they can be evoked by different stimuli.

It has also been pointed out before that colour perception is largely independent of retinal position, when targets are scaled to match the local scale of the visual field (Noorlander *et al.* 1983; Block 2007; Tyler 2015; Haun *et al.* 2017). One counterargument to this is that objects in natural scenes, as opposed to eccentricity-scaled experiment stimuli, do not change size when they fall at different retinal eccentricities, and so the size dependence of colour perception across the retina is not relevant to natural vision (Van Gulick 2007; Burnston 2018). However, natural scenes are scale invariant (Burton and Moorhead 1987; Ruderman and Bialek 1994), meaning that, on average, any location within a scene may contain spatial content at all scales. So, a neuron with a large colour-opponent receptive field in the periphery is as likely as one with a small foveal receptive field to find a stimulus that excites it.

### Caveats and conclusion

The spatial vision model I have used in this study is not unique. There are many alternate formalisms for sensitivity across the visual field (e.g. Watson and Ahumada 2016; Schütt and Wichmann 2017; Watson 2018), sensitivity to different levels of contrast (e.g. Lu and Dosher 1999), and colour vision. There are also many alternate models of blur perception, only some of which would be compatible with the contrast response model I have presented (e.g. Field and Brady 1997). I selected the components of this model for their simplicity and compatibility. The important thing for my purposes is that these different models are generally *psychophysically* equivalent. That is, I expect that an alternate spatial vision model could be constructed, but as long as it fits the psychophysical properties listed in points i-x (Section Methods), the ensuing statistical analyses will be the same. So, I do not believe that the results of this study are the consequence of some peculiar modelling choices.

I hope the scope of these findings is clear. The familiar psychophysical patterns of exponential decline in sensitivity with eccentricity and spatial frequency, and the steeper decline for chromatic channels, do not mean that peripheral vision is incapable of representing colour or sharpness in the same way as foveal vision. This is not to say that capacity for visual qualities *must* be represented in the same way across the visual field. The main analyses of colourfulness and sharpness are merely describing the informational relationship between the visual system and complex scene stimuli. How the visual system uses this information to form higher-level representations is a question that could be addressed with the suprathreshold regime of a model like what I have presented, but except for the perceived blur model, I have not tried to do it in this study (for reasons detailed in the Appendix, I expect the model would need more work).

Even given that the visual system has the necessary information for representing colourfulness and sharpness across the visual field, there are other processes that may interfere. Crowding is an obvious difference between foveal and peripheral vision, but it is unknown how crowding interacts with apparent colourfulness or sharpness. Attention is another obvious difference between the foveal and peripheral fields, since it naturally resides at the foveal field, but this has already been addressed to some extent (Section Sharpness): withdrawing attention from a region of the visual field does not result in a collapse of our capacity to represent colour and contrast. Rather, withdrawing attention results in a modest decrement in that capacity. The phenomenon of inattentional blindness, where unattended objects or features (or their absence) go completely unnoticed, might have little to do with the effects of attention on low-level visual perception. Instead, the phenomenon might be more similar to inattentional *agnosia* than blindness (Simons 2000; Koivisto and Revonsuo 2008): just as an individual with object agnosia experiences colours and textures without experiencing the object those qualities compose (Farah 2004), we might routinely experience the spatial qualities of an unattended peripherally viewed scene, without recognizing what they compose. Given that the known effects of attention on contrast perception are rather moderate, I take this to be a simpler alternative than supposing that attention might be necessary, through the action of some as-yet unknown mechanism, for the experience of colour (as considered in e.g. Cohen *et al.* 2020). However, the present results are not evidence against such a mechanism, and such a powerful ‘refrigerator light phenomenon’ would be, by definition, very difficult to test experimentally.

A goal of this study was to dispel the notion that peripheral experience of colour and sharpness must be illusory because the periphery is unable to support such percepts. Given the results of the current study, is there still any sense in which the qualities of peripheral vision might be thought of as illusory? I think there certainly is. In one study (Balas and Sinha 2007), it was found that observers judge rapidly presented scenes to be in full-colour even when a significant portion of the scene area is fully desaturated. If we take this result at face value, supposing that the local spatial structure of the scene actually became colourful as a result of, for example, some top-down influence, it is a proper visual illusion: the scene appears one way, even though the stimulus would have been expected to elicit a different appearance. The ‘uniformity illusion’ (Otten *et al.* 2017) is similar, except with textures rather than colours: parts of the display appear one way, though the stimulus is a very different way. These are illusions in that the percept is at odds with the stimulus (or with our expectations of how it should appear). That the appearances are real, that they feel the way they seem to, is well-within the capacities of the visual system. If ‘illusion’ is taken to mean that one believes one experiences something that one *cannot* experience, then there is nothing obviously illusory about the apparent colourfulness and clarity of a natural scene that fills the visual field.

## Supplementary Materials

Code implementing the spatial vision model, references to the scene images used in this study, and other materials are found online at https://osf.io/8xf9w/.

Supplementary data is available at *NCONSC Journal* online.

## Footnotes

1 This implementation of the B/Y channel ignores the fact that human spectral sensitivity is far better for yellow spots than for blue spots (Abramov & Gordon, 1977). The human visual system is not so simple as to symmetrically encode blue and yellow, but the model is, so it tends to underestimate yellow and overestimate blue contributions to stimuli. A similar problem exists for the achromatic channel, in that true human sensitivity to ‘dark’ spots is generally better than for ‘bright’ spots (Haun & Peli, 2013b; Whittle, 1992), while the model makes these symmetrical. Psychophysically, green and red are *relatively* more symmetric, though see point xxi in the appendix 1.

2 Why not tie apparent sharpness simply to the responsiveness of the smallest filter at each position? There are some image features, especially fine-grained textures, that will not be judged as ‘sharp’ under the FC metric, but would be if the metric were relaxed to extend only over the finer (or finest) filter scales. However, the model would then be inclined to judge features as sharp erroneously, such as when white noise is added (as in (Kayargadde & Martens, 1996; Kurihara et al., 2009)), even though adding white noise to edges in natural scenes tends to reduce their apparent sharpness ((Kurihara et al., 2009); perhaps as a consequence of ‘coarse-scale suppression’ as reported in (Haun & Hansen, 2019; Haun & Peli, 2013b)). Considering these issues, the broadband FC metric is the more conservative choice: it should underestimate, rather than overestimate, apparent sharpness.

## Appendix

### Some notable spatial vision properties left out of the model

These properties are perhaps pertinent to the question of colour and sharpness percepts across the visual field, but they are left out of the model in this paper. Many of these amount to aggregations of contradictory interactions (i.e. of facilitation and suppression), so we can think of them as ‘washing out’ (e.g. xii, xiii). Others are factors that, by their omission, mean the model is probably underestimating rather than overestimating sensitivity (e.g. xvii, xix, xx). And some are just too complex and would not yield worthwhile returns on the questions at hand (e.g. xxi). However, these are significant aspects of spatial vision psychophysics that touch on the questions at hand, and their inclusion would improve the model, so their omission should be acknowledged:

xi. The temporal aspect of contrast sensitivity is ignored. The model’s sensitivity is effectively based on the typical 100–500 ms pulse-windowed targets in many traditional trial-based psychophysics tasks (i.e. temporally broadband and generally low-pass or low-frequency-biased). This can be reasonably linked (as in Haun and Peli 2013b) to the typical duration of fixations between saccades (Henderson 2003).
xii. Orientation anisotropies [oblique effects and horizontal effects (Essock *et al.* 2009) and radial anisotropies (Westheimer 2003), and so on, of various types are small and ignored here.
xiii. Surround-modulation and other lateral-interaction effects (Yu *et al.* 2003; Chen and Tyler 2008) are ignored.
xvi. Interactions between the colour channels (e.g. Kim and Mullen 2016) are ignored.
xv. Contrast adaptation effects are ignored.
xvi. The low-spatial-frequency plateau in contrast sensitivity has a form that I did not attempt to match closely.
xvii. Cross-orientation masking is dependent on stimulus parameters and is usually weaker than self(same-orientation)-masking (Foley 1994; Meese and Holmes 2007), but I set it to have constant strength equivalent to the strength of a filter’s self-masking, i.e. =1. Thus, the model always overestimates the strength of cross-orientation masking.
xviii. Cross-frequency interactions are captured only by the cross-frequency norm (Methods), ignoring specific patterns of interaction that suggest that the visual system works to reduce the representation of lower frequency content in the presence of higher frequencies (Haun and Essock 2010; Haun and Peli 2013b).
xix. Contrast sensitivity is known to improve on virtually every dimension when the scene’s luminance is increased, and natural scenes generally have far higher luminances than experimental contrast displays; the current model is fixed to the low-luminance, but still photopic, experimental context (so sensitivity may generally be underestimated by the model).
xx. Binocularity adds many complexities to spatial vision. The contrast sensitivity data that the model is fitted to was all collected monocularly; binocular sensitivity and discrimination is generally better than monocular (Campbell and Green 1965; Legge 1984), so the estimates of sensitivity can generally be considered underestimates on this point.
xxi. When we look closely, colour perception across the visual field has much more inhomogeneity across the visual field than the model would suggest. For example, in many - but not all - observers ‘green’ percepts are gradually attenuated towards the periphery (Stromeyer *et al.* 1992), while R/G contrast sensitivity paradoxically continues to follow the expected pattern. There are also reports that colour stimuli can appear *more saturated* in the periphery than at the fovea (e.g. Vanston and Crognale 2018). Also, in the fovea there are no S-cones and so foveal sensitivity to blue spots is very poor (Wald 1967), although foveal blue *percepts* still are possible (Magnussen *et al.* 2001). See also footnote 1 in Section Filter Structure regarding asymmetries within each colour channel; in my model, each colour is encoded symmetrically with its opponent, which is a nontrivial simplification.
